# Pan-Genome Analysis of Human Gastric Pathogen *H. pylori*: Comparative Genomics and Pathogenomics Approaches to Identify Regions Associated with Pathogenicity and Prediction of Potential Core Therapeutic Targets

**DOI:** 10.1155/2015/139580

**Published:** 2015-01-29

**Authors:** Amjad Ali, Anam Naz, Siomar C Soares, Marriam Bakhtiar, Sandeep Tiwari, Syed S Hassan, Fazal Hanan, Rommel Ramos, Ulisses Pereira, Debmalya Barh, Henrique César Pereira Figueiredo, David W. Ussery, Anderson Miyoshi, Artur Silva, Vasco Azevedo

**Affiliations:** ^1^Atta-ur-Rahman School of Applied Biosciences (ASAB), National University of Sciences and Technology (NUST), Islamabad 44000, Pakistan; ^2^Laboratory of Cellular and Molecular Genetics, Federal University of Minas Gerais (UFMG), 31907-270 Belo Horizonte, MG, Brazil; ^3^Department of Bioinformatics, Mohammad Ali Jinnah University (MAJU), Sehala Road, Islamabad 44000, Pakistan; ^4^KIMS, Khyber Medical University, Peshawar 25000, Pakistan; ^5^Federal University of Pará, 66075-110 Belém, PA, Brazil; ^6^Laboratory of Aquatic Animal Diseases (AQUAVET), Department of Preventive Veterinary Medicine, Federal University of Minas Gerais, 31907-270 Belo Horizonte, MG, Brazil; ^7^Centre for Genomics and Applied Gene Technology, Institute of Integrative Omics and Applied Biotechnology (IIOAB), Nonakuri, Purba Medinipur, West Bengal 721172, India; ^8^Centre for Biological Sequence Analysis (CBS), Technical University of Denmark, 2800 Kongens Lyngby, Denmark

## Abstract

*Helicobacter pylori* is a human gastric pathogen implicated as the major cause of peptic ulcer and second leading cause of gastric cancer (~70%) around the world. Conversely, an increased resistance to antibiotics and hindrances in the development of vaccines against *H. pylori* are observed. Pan-genome analyses of the global representative *H. pylori* isolates consisting of 39 complete genomes are presented in this paper. Phylogenetic analyses have revealed close relationships among geographically diverse strains of *H. pylori*. The conservation among these genomes was further analyzed by pan-genome approach; the predicted conserved gene families (1,193) constitute ~77% of the average *H. pylori* genome and 45% of the global gene repertoire of the species. Reverse vaccinology strategies have been adopted to identify and narrow down the potential core-immunogenic candidates. Total of 28 nonhost homolog proteins were characterized as universal therapeutic targets against *H. pylori* based on their functional annotation and protein-protein interaction. Finally, pathogenomics and genome plasticity analysis revealed 3 highly conserved and 2 highly variable putative pathogenicity islands in all of the *H. pylori* genomes been analyzed.

## 1. Background 

The genus* Helicobacter *includes bacterial species which colonizes the gastrointestinal tract (GIT) of human and other mammals. Both* H. pylori *and non-*pylori *species in the genus are involved in gastrointestinal diseases [1, 2]. In humans,* H. pylori *causes diseases like gastritis and peptic ulcers, which can lead to the development of gastric cancer (~10% of cancer deaths around the globe). Therefore, the pathogen was enlisted as a class I carcinogen by WHO in 1994 [2, 3].

The infection rates remain as high as 90% in developing world, and in low socioeconomic regions like the ones located in Africa, East Asia, and Central America the diseases (peptic ulcer and gastric cancer) are highly prevalent. However, in the developed world and industrialized cities the prevalence is considerably lower, most probably due to efficient preventive and public health measures taken by some western countries [4].

Previous studies indicated that most of the* H. pylori *isolates could be classified either by sequence diversity or gene content. This is the main reason of getting certainly great differences in isolates of* H. pylori *strains from different continents. These genomic variations are indicative of genetic drift during geographic isolation, adaptation, and coevolution of the pathogen within different ethnic groups of human population [5, 6].

The mechanisms of* H. pylori *pathogenesis are relatively complex than other bacterial strains; however, some well-known factors such as the type IV secretion system (T4SS) and the CagA oncoprotein have been demonstrated to play an important role in inflammation and carcinogenesis of GIT [7, 8]. Beside these, there are few other virulence factors which have been identified and discussed in the literature. These factors help in identifying the infectious agents and disease causing behavior of the said bacterial specie.* H. pylori *has a diverse genome and carries many plasticity zones which differ among strains. Thus, the identification of these and other novel regions in a particular strain would offer insights into the pathogenic diversity of* H. pylori *strains [9]. A detailed phylogenomics and comparative genomics study is required to understand the association among different isolates and disease symptoms.

To explore the conserved features and shared genomic contents, Salama et al., in 2000, attempted to determine the core set of genes in 15* H. pylori *strains by microarray method and observed that 1,281 genes are shared by all the examined strains. However, the examined strains were mainly isolated from western countries [10, 11]. For detailed view of the diversity and conservation in* H. pylori *strains, Gressmann et al., in 2005, determined the core genome of a larger set and global representative strains and suggested that* H. pylori *core genome consists of 1,111 genes [6].

These findings indicated that as the number of genome sequences increases, the actual or true core genome can be obtained; furthermore, the genomic diversity may also be predicted. Among the genetic repertoire of the* H. pylori *including genes for various functions, the core minimal set of genes is of particular interest. It is required for maintaining the basic cellular life and is indispensible for survival of the organism [12]. Calculating the conserved/core genome of diverse strains of* H. pylori *at larger scale would provide an overview of strain diversity and this data could be utilized for further use in diagnostics and therapeutics applications.

Furthermore, the identification and characterization of core essential genes, in other words the minimal essential genes set, in* H. pylori *species would be an interesting strategy both theoretically and experimentally to understand the basic requirements for cellular life. A practical example of the importance of minimal gene set characterization is in drug development. Drugs are usually designed against essential cellular processes and essential gene products of microbial cells as they are specific and provide promising new targets for such drugs [13].

Recently, we witnessed an increase in the number of complete genome sequences of* H. pylori *on public databases. With this, we intended to follow reverse vaccinology strategy to predict the potential core-immunogenic, virulence factors which can serve as putative vaccine candidates against* H. pylori* and, moreover, to utilize all those available complete genome sequences to get insights into the genomic diversity in* H. pylori *species and to compare the genomes. We believe that comparative analysis of this pathogen (*H. pylori*) genome sequences and their nonpathogenic relative will offer a unique opportunity for the identification of regions of genomes such as novel pathogen specific pathogenicity islands [9, 14, 15].

## 2. Material and Methods

### 2.1. Data Collection and Management

The gastric pathogen* H. pylori *have multiple complete genomes available on public databases for scientific exploitation. Here, we selected a total of 39 complete* H. pylori *genomes (available at the time of analysis). The draft or incomplete genomes were not included for instance, to have uniformity in the analyses. GenBank (gbk) files were obtained from NCBI genome browser (http://www.ncbi.nlm.nih.gov/genome/browse/). As a starting point, DNA sequences in a fast format were extracted from all the GenBank files (chromosome and plasmid sequences were merged, as they were required) and then subjected to program* RNAmmer* [16] for prediction of full length 16S rRNA gene sequences. The program* Prodigal*, a gene finding algorithm, was also used to predict open reading frames (ORFs) in all genomes [17]. We, however, have seen slight variations in the number of genes/ORFs in our predicted ones and those available publicly (GenBank). To avoid inconsistencies and to get uniformity in the resultant data, we used a single gene finding program.  Table 1 demonstrates the basic genome information, that is, size in base pairs, number of predicted proteins, chromosome/plasmid accession numbers, and percent AT content.

### 2.2. Phylogenetic Analysis

In order to better understand the evolutionary relationships and genomic variations at genus level, all 39 sequences genomes of* H. pylori *species were analyzed for 16S rRNA genes using* RNAmmer* [16]. Multiple sequence alignment of all obtained 16S rRNA genes was aligned using* ClustalW* [18]. Sequences generated with score >1700 are usually considered to be more reliable and necessary for phylogenetic analysis [16, 19]. Phylogenetic tree was generated following Neighbor Joining algorithm [20] by using* MEGA6* [21].

### 2.3. Proteome Comparisons (Pairwise Alignment)

All the selected* H. pylori *genomes were translated into their proteomes. BLASTp comparisons were carried out for all the proteins present in one genome against all the proteins in the other genomes in the study [19, 22]. The BLAST parameters were set as follows: BLAST cutoff *e*-value was 1*e* − 5; homologs were selected at *e*-value of 1*e* − 8; identity homolog was 0.95; the blast minimum score was 30; and minimum identity was 90. Reciprocal best blast hits were selected for generation of blast matrix which is shown in Figure 2. Each cell in the blast matrix demonstrates genes/proteins (*x*-axis) reciprocal hits with respect to the genome listed to the right (*y*-axis). Each corresponding rectangular box (between any two genomes) in the matrix represents the number of reciprocal hits (shared proteins) and the total number of proteins. For example, two genomes, G1 and G2, will lead to (G1/G2)∗100 = X% of the proteins/genes in G1 had reciprocal hits, while (G2/G1)∗100 = X% of the proteins/genes in G2 had reciprocal hits. The diagonal row of boxes indicates the internal homologies against itself (genome). The colored matrix is generated for all the 39 genomes/proteomes with the scale given, indicating the relative homology between corresponding genome/proteome.

### 2.4. Pan-Genome Analysis and Characterization of Core Genome

The* H. pylori *conserved core families (CGFs) and pan-genome families (PGFs) were estimated followed by previously established method [19, 22]. CGFs and PGFs were estimated by employing single-linkage clustering on top of BLASTp alignments, with the notion that any two genes in the data set are considered to belong to the same gene family and should be considered as “conserved” if their amino acid sequences are at least 50% identical over at least 50% of the length of the longest gene [22, 23]. The strategy may also be termed as 50/50. Doing so, multiple genes may belong to single gene family and the number of gene families would be lower than the actual number of genes in a genome. Those genes which do not fit to the criterion would constitute individual genes families. Gene families with at least one gene in common were gathered into the core genome. The rest, either unmatched or not qualifying according to the criterion, constitute the species pan-genome [24–26].

### 2.5. Essential Gene Families and Nonhost Homologs Prediction

The* H. pylori *predicted core genome was then aligned with the Database for Essential Genes, DEG (http://tubic.tju.edu.cn/deg/), for estimation of essential core gene families, EGFs [12]. DEG contains essential genes data from 32 bacteria, including* E. coli, B. subtilis, H. pylori, S. pneumoniae, M. genitalium, *and* H. influenzae. *The BLAST comparison settings for selecting the essential genes/proteins were followed as previously described [27]: expected value (*e*-value) cutoff of 10^−10^; percentage of identity ≥ 35% between query and hits; and minimum bit score of 100. In the case of* H. pylori*, the host (human) genome/proteome was downloaded from NCBI (*taxid*: 9606) and BLASTp analysis were performed. In BLASTp comparison parameters, the percentages of identity and *e*-value were kept <35% and 0.005, respectively. Proteins without hits below the *e*-value inclusion threshold were collected as nonhost bacterial proteins and called nonhost essential gene families (nHEGFs).

### 2.6. Screening Core Exoproteome

For prediction of the potential immunogenic and vaccine candidate genes and proteins, the predicted nHEGFs (283) were analyzed for their surface localization and signal peptide detection using CELLO (subCELlular LOcalization predictive system; http://cello.life.nctu.edu.tw/) and PSORTb (http://www.psort.org/psortb/). Exoproteome and secretome always remain a good source for therapeutic targets. Moreover, for the prediction of potential vaccine targets and analyses, proteins were analyzed for their transmembrane helices, MHC class I and II binding epitopes, nonhost conservation, and pathogen specific proteins.

### 2.7. Prioritizing Vaccine Targets

Proteins falling in nHEGFs category were then analyzed for their potential as therapeutic targets. Proteins having <110 KDa are considered to be good therapeutic targets. Proteins labeled as surface or secreting proteins predicted from CELLO and PSORTb in previous step were subjected to ExPASy PI/MW tool [28] which calculated their molecular weight.

### 2.8. Functional Annotation of Targets

Predicted surface and secreting proteins were then analyzed for functional annotation using Blast2Go [29] which is a workstation for functional annotation of nucleotide/protein sequences; the program not only provide annotated results but also give remote access to user which help in analyzing data with different graphical and statistical functions.

### 2.9. Protein-Protein Interactions (PPIs) of Prioritized Targets

Proteome-scale interaction analysis of prioritized proteins was performed using Search Tool for the Retrieval of Interacting Genes (STRING) (http://www.string-db.org) [30]. STRING being a comprehensive database and user friendly tool provides a good opportunity of protein-protein interactions along with their available information regarding functional categorization and domain evaluation, and so forth.

### 2.10. Epitope Mapping

To predict best vaccine targets prioritized proteins with their full length sequence were subjected to BCPred analysis [31] to predict B-cell epitopes. Cutoff score for 20-mer epitope prediction was maintained as 0.8 and epitopes having >0.8 value were then subjected to VaxiJen [32] to check antigenicity (threshold = 0.4, ACC output).

### 2.11. Pathogenomic Analysis and Prediction of Pathogenicity Islands

In order to identify pathogenicity islands (PAIs) in* H. pylori *species, we have performed an analysis with PIPS (Pathogenicity Island Prediction Software) [33]. PIPS identifies PAIs according to their main feature: genomic signature deviations, that is, G + C content and codon usage deviations; presence of virulence factors, transposase genes and flanking tRNAs genes; and absence in nonpathogenic organism from the same genus or related species. The analyses were performed on* H. pylori *strains 26695, Cuz-20, J99, PeCan4, and SouthAfrica7, which are representative of Europe, East Asia, West Africa, South America, and South Africa, respectively. Additionally,* Wolinella succinogenes *DSM 1740 was chosen as nonpathogenic closely related species for PIPS analysis requirement [34]. After prediction step, the sizes of all PAIs in all 5* H. pylori *genomes used in this step were manually inspected through graphical genome comparisons in ACT: the Artemis Comparison tool [35]. Finally, we obtained reference PAIs from the genomes which can be used as representative islands among all. The reference PAIs were then compared with all 39 genomes of* H. pylori *included in this study using the proteome comparison data and plotted a heatmap for easy visualization of percentage identity.

## 3. Results and Discussion

### 3.1. *H. pylori *Genome Statistics and Features

*H. pylori *is a Gram-negative microaerophilic bacterium which colonizes the human stomach, a pathogen belonging to epsilon-bacteria. [1, 36, 37].* H. pylori *species has a total of 39 complete genomes sequences on NCBI GenBank (at the time of analyses). The majority of them are in Refseq. Of the 39* H. pylori *complete genomes, about half of them (17) carry plasmid DNA (one or more) (Table 1). The total number of proteins in 39* H. pylori *genomes was calculated as 59,958, while an average* H. pylori *genome was found to contain 1,537 protein coding genes (CDS). The genome sequencing of this important pathogen started with* H. pylori *26695, which was obtained in 1997. It has been observed that it contains a circular chromosome of 1,667,867 base pairs with an average GC content of 39% [36]. Later on, while other genomes were sequenced it became prominent that* H. pylori *genomes have uniform (lower) GC contents. According to GenBank data, the %GC does not have considerable variations and range from 38 to 39% (http://www.ncbi.nlm.nih.gov/genome/browse/). During the analyses of* H. pylori *genomes, an average 61% of AT content was observed. The closely related strain J99 isolated from an American patient having duodenal ulcer; when compared to* H. pylori, *26695, a total of 1,406 genes were found common amongst them. The J99 was observed to have 86 unique genes; however, both strains share the important* cag *pathogenicity island, which codes for the type IV secretion system, facilitating the transport of* Cag*A cytotoxin to gastric epithelial cells [3, 38]. Later, the strain HPAG1 (isolated from an elderly women in Sweden) sequenced in 2006 [39] was compared to the previous two strains (J99 and 26 695) and it has a smaller genome size and shared the* Cag*A and* vac*A (virulent allele). HPAG1 was found to contain 43 strain specific genes. Another strain, G27, which is similar in size (1652983 bp) to the previous strains (26 695, J99, and HPAG1), contains a plasmid (11 genes) and 58 specific genes. However, the* cag *island is reported to be disrupted by a transposon in G27 [3, 8]. The strain Shi470 has a genome relatively larger in size (~1.6 Mbp) than the previous strains. The recently sequenced genome* H. pylori *Sahul64 (ALWV01) isolated from an Australian Aboriginals also lies in the range and has a genome 1.64 Mbp consisting of 1579 predicted genes [40]. However, there is a consensus in the scientific community that there is an increase in* H. pylori *genomic variability and the phenomena of mutations and recombination are continuously occurring. The analyzed genomes in this study and their genome statistics, shared genes/proteins, and genes associated to particular strains were predicted and are shown in Table 1. For example, the highest number of strain specific genes families (189 GFs–207 genes) was observed in the* H. pylori *strain 26695 while the lowest (6 GFs) was found in* H. pylori *Shi169.

### 3.2. Phylogenetic Analysis of Ribosomal RNA

**Figure 1:**
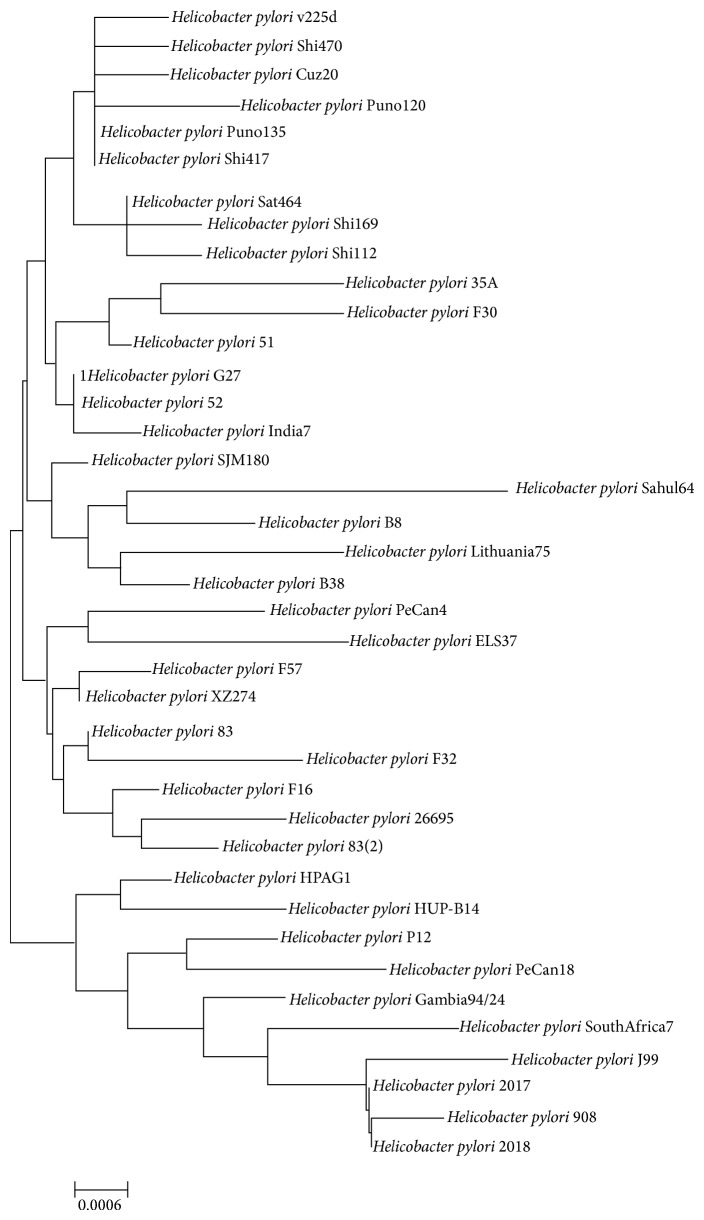
The evolutionary history inferred by Neighbor Joining method (NJ).* MEGA6* was used for multiple sequence alignment and construction of phylogenetic tree of all 39* H. pylori *strains. Branch lengths were computed using evolutionary distances generated by Maximum Composite Likelihood method.

*H. pylori *is a well-recognized human gastric pathogen, being colonizing the earliest human populations and it is believed to have coevolved and comigrated with its hosts across the world [4]. A phylogenetic analysis has been done for 39* H. pylori *species to understand pattern of evolution and distribution of the organism. The phylogenetic tree based on 16S rRNA was generated from the resultant data (extracted from genomes) using* MEGA6* [21]. Neighbor Joining method was employed to construct the tree [41]. After generating possible pairwise and multiple alignments* MEGA6* illustrates a phylogenetic tree representing the optimal tree with the sum of branch length = 0.04530419. The tree is drawn to scale, with branch lengths in the same units as those of the evolutionary distances used to infer the phylogenetic tree (Figure 1). The evolutionary distances were computed using the Maximum Composite Likelihood method [42] and are in the units of the number of base substitutions per site. The analysis involved 39 nucleotide sequences. Codon positions included were 1st + 2nd + 3rd + noncoding. All positions containing gaps and missing data were eliminated. There were a total of 1482 positions in the final dataset. A newly sequenced strain (Sahul64) isolated from Australian Aboriginals [40] has a draft genome available and incorporation of this genome in phylogenetic analysis shows maximum branch length in its clade representing the higher evolutionary time. The previous reports have revealed that the* H. pylori *species divides into two clusters: one with the species which colonize the stomach of mammals (so called gastric species) and the other with species that inhabit the intestine and biliary tracts (so called enterohepatic cluster) [5]. To better understand intraspecies relationships in* H. pylori *the amount and percentage of shared proteome are predicted.

### 3.3. Proteome Conservation in* H. pylori *Strains (Pairwise Alignment)

**Figure 2:**
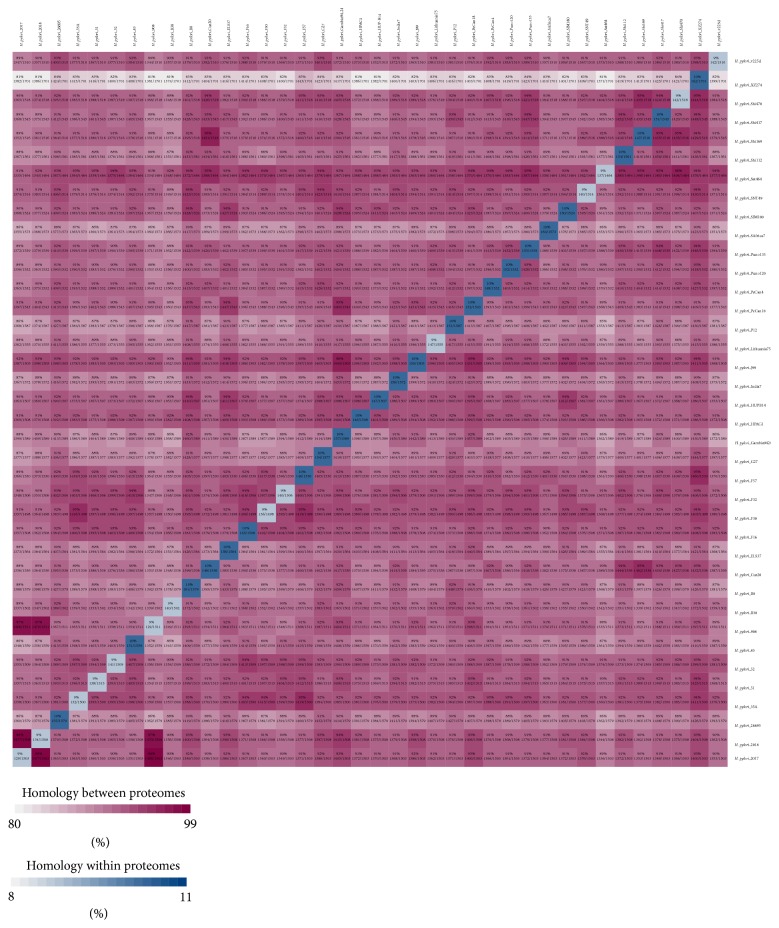
Whole genome/proteome pairwise alignment and comparative analysis. The translated genomes of all* H. pylori *available strains are analyzed by BLASTp analysis. Pairwise comparisons across* H. pylori *proteomes are plotted in blast matrix. The shared proteome between any two* H. pylori *genomes and the percentage of similarities is calculated and shown in corresponding boxes, where the color intensity indicated the similarity. The diagonal row of rectangular boxes in the matrix illustrates the internal homology against its own proteome.

We observed higher similarities in genomic contents of* H. pylori *strains in phylogenomic analysis; therefore, we decided to compare the whole predicted proteome (proteins) of all* H. pylori *strains to estimate the amount of proteins they share. The* H. pylori *genomes were pairwise compared (BLASTp). To estimate the amount of shared proteome between and among strains, a matrix is generated from the data of comparisons which is shown in Figure 2. Both number and percentage of shared proteome (proteins) are shown in corresponding box of the matrix. For example, we can see in the color matrix a maximum proteome conservation of ~98% and a minimum of ~81%. The maximum similarity we observed is in* H. pylori *2017 and* H. pylori *2018 and vice versa. Inversely to this, one can see the minimum ~81% proteome similarity between* H. pylori *2018 and* H. pylori *Shi 470. Similarly,* H. pylori *908 and* H. pylori *B8 share the same amount of proteins with* H. pylori *Shi 470. These findings are interesting when compared with phylogenetic trees and reflect the consistent interstrain relationships. For example,* H. pylori *2017 and* H. pylori *2018 are located quite close in phylogenetic tree; this also reflects the similar geographic relationship (West Africa). Similarly, they are closely related to* H. pylori 908*, isolated from an African patient living in France having history of recrudescent duodenal ulcer disease, which in turn was found phylogenetically related to* H. pylori *J99, isolated from gastric carcinoma and gastritis patients in Costa Rica. These findings helped in understanding the intraspecies (strains) diversity and/or similarity and its correlation with that of phylogenomic variations based on genomic contents.

### 3.4. Pan-Genome Analysis of* H. pylori*

**Figure 3:**
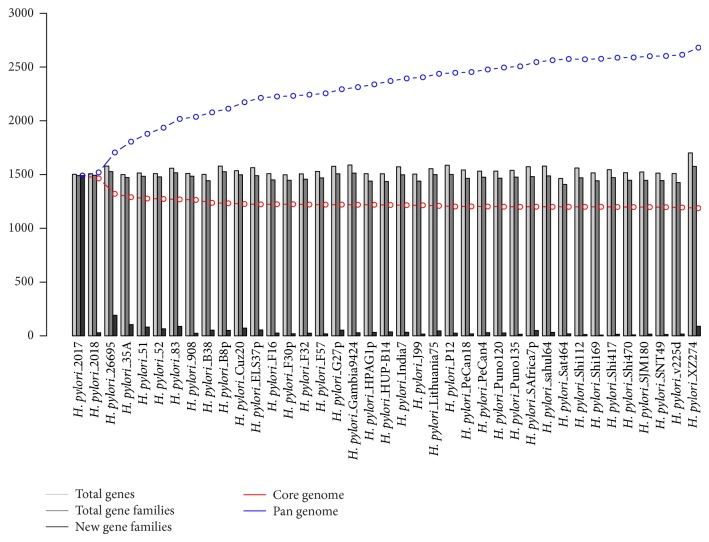
Core and pan-genome estimation for the genus,* H. pylori* and non-*pylori* species. The figure demonstrates the distribution of core (1,193) and pan-genome (2,614) of* H. pylori *species (Table 1). The pan-genome plot represents total number of genes, gene clusters (families) for each genome (light grey), new gene families (dark gray) pan-genome (blue), and core genome (red). Name of genome is provided on *x*-axis and number of genes can be observed on *y*-axis.

The global gene repertoire of 39* H. pylori *genomes comprises 59,958 genes. Among this set of genes, core or central genome was estimated to contain 1,193 CGFs and the pan-genome was found to contain 2,614 PGF. An average genome of* H. pylori *contains ~1537 genes (the lowest 1,464 in* H. pylori *Sat464 and the highest 1,701 in* H. pylori *XZ274). The calculated core genome represents ~77% of the average genome size (~1,537 genes/proteins). As expected, the* H. pylori *genomes (isolates) share a large part of their genomic content and a decrease in the number of new GFs/genes was observed as compared to what was accumulated upon subsequent genome inclusion to the study; this can be seen in the pan-core genome plot in Figure 3. We also observed accumulation of an average of ~39 new gene GFs (40 genes) with subsequent addition of genome; however, the highest numbers of species specific (unique) GFs observed were 189, 103, and 89 in* H. pylori *26695,* H. pylori *35A, and* H. pylori *XZ274, respectively (Table 1). These findings demonstrated that these particular strains are more diverse among those analyzed strains; the phylogenic tree also reflects similar image of evolutions or genome distributions. The pan-genome, on the other side, rose at a lower rate and roughly twice the size (~57%) of the core genome while the core genome is about 45% of the global gene repertoire, that is, the pan-genome of* H. pylori*. In a previous study by Salama et al., in 2000, they determined the core set of genes in 15* H. pylori *strains with a microarray method, and a total of 1,281 genes were found to constitute the core genome [11]. As the strains were mainly isolated from only the western part of the world, it was difficult to estimate that those core genes would also represent the larger number of species, once they became available. However, it is not much surprising to estimate high number of CGFs for a few intraspecies genomes due to higher genomic similarities. On a random comparison, the first 15 genomes in our dataset represent 1,223 CGFs, which is close to the core genome (1,281) previously observed. Furthermore, 25 genomes were found to share 1,208 CGFs and the total set of 39 genomes constitutes a core genome of 1,193 CGFs (Additional File 1, in Supplementary material available online on http://dx.doi.org/10.1155/2014/139580). Our findings are quite closed to previous data, indicating the consistent behavior, evolution, and gene distribution [11, 40]. Nevertheless, we are sure that even with the addition of more genome sequences, the pattern would remain the same or the conserved regions would be limited, but not necessary. Also, an average of ~40 new genes added to the study on addition of each new strain. The overall picture of* H. pylori *genomes statistics (genes, gene clusters (GFs), and unique genes associated with), core, and pan-genome pattern is shown in Figure 3, and the individual genomic data can be found in Table 1.

### 3.5. *H. pylori *Core EGFs and Nonhost Homologs Predictions

Essential genes in a genome are the genes which are crucial for growth, cellular activities, and foundation of life. These genes represent a minimal gene set which is essential for a living cell. The identification of essential genes is of practical importance in drug designing against bacterial infections, and due to the fact that most of the antibiotics target essential cellular processes and essential gene products of microbial cells, they are suitable candidate targets for such drugs [43]. For this propose, we subjected our predicted CGFs (1,193) against the database of essential genes, DEG and classified CGFs as EGFs if they had significant similarity against experimentally validated essential genes in other bacteria, bacterial group, or database. We predicted a total of 779 (~65%) EGFs in our core data set with the following selection parameters: *e*-value cutoff 10^−10^, percentage of identity ≥ 35% between query and hits, and a minimum bit score of 100 [27]. The list of those 779 EGFs along with their matching DEG ID is provided in Additional File 2. In principle, ideal targets for bacterial therapeutics are pathogen's genes/protein whose homologs are absent in the corresponding host. In the case of* H. pylori, *colonizing humans, we downloaded the human proteome (*taxid*: 9606) available on GenBank, which has 34,521 proteins. BLASTp analyses were performed and the predicted core EGFs in previous step (779) was aligned against human proteome. This time, the *e*-value cutoff was set to 0.005 and the percent of identity to 35%. Proteins without hits below the set parameters were chosen as nonhost bacterial proteins. Two datasets were then generated and the so-called pathogen essential gene families having human homologs (497) were kept aside. The remaining 283 core essential proteins were found specific to pathogen (*H. pylori*) and do not have corresponding human homologs with same threshold (Additional File 3). The later set (core nHEGFs = 283) were selected as potential candidates and subjected to further validations (sequence and structural).

### 3.6. Vaccine Candidates Identification

For multiple related pathogenic species (intraspecies), once the complete genome sequences are available, it is a desirable task to predict global vaccine targets that are conserved among all genomes. Generally, surface and secreted proteins are involved in bacterial pathogenesis and are recognized as good candidates for vaccine development [19, 44]. On the other hand, cytoplasmic and inner membrane proteins may not be good candidates for vaccine development; however, they can be targets for drug designing against bacteria. The following steps have been followed for the prediction of potential candidates.

### 3.7. Core Exoproteome Prediction

For* H. pylori *global vaccine identification, we analyzed our predicted nHEGFs set of EGFs (283) by an online protein localization tool CELLO [45]. Among the 283 (nHEGFs), 29 proteins predicted to be associated with bacterial cell surface (7 outer membrane, 12 inner membrane 8 periplasmic, and 5 extracellular) and some of the proteins were found to have multiple locations. Multifasta file containing predicted surface proteins (sequences) is created and is provided as Additional File 4.

### 3.8. Prioritization of Vaccine Targets

The predicted 29* H. pylori *(CGFs/EGFs) surface proteins predicted by using* CELLO* [45] and* PSORTb* [46] were then analyzed for their molecular weight. Proteins having molecular weight <110 KDa are thought to be good targets for vaccine. nHEGFs generated in our analysis showed that 28 out of 29 proteins have molecular weight <110 KDa which are then supposed to be good targets for vaccines (Table 2).

### 3.9. Functional Annotation of Targets

**Figure 4:**
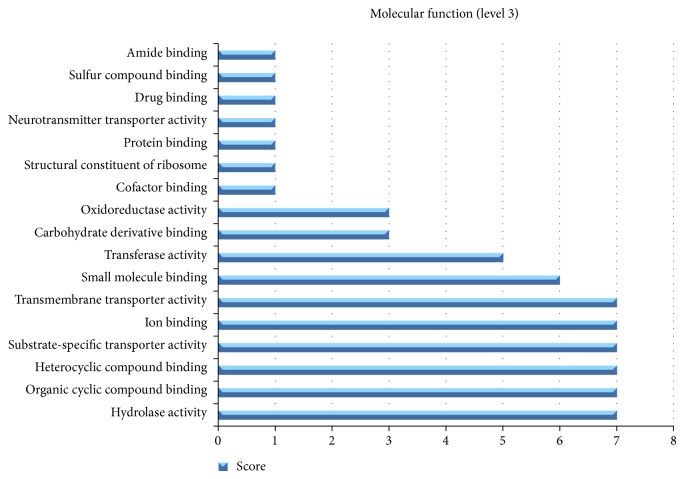
Functional characterization of proteins selected as potential vaccine targets. Total of 29 nonhuman homolog proteins were functionally annotated using* Blast2go* and distribution of their molecular functions in analyzed in the form of graph.

Blas2go being a reliable workstation was used to generate functional annotation of targeted proteins [29]. Proteins were analyzed for their functions, characterization as enzymes, and InterPro scan. 28 out of 29 proteins were predicted to be potential therapeutic targets and are found to be involved in many essential biological functions such as abc transporter, membrane protein, intertase, and potassium transporter. Eleven core proteins are enzyme in nature and thus can be the best for vaccine or therapeutic targets. Complete functional annotation of proteins is given in Table 2. Blast2go was used to analyze molecular and biological function distribution among 28 proteins and a graph was obtained showing most of the proteins involved in hydrolase activity, organic cyclic compound binding, heterocyclic compound binding, substrate-specific transporter activity, ion binding, and transmembrane transporter activity (Figure 4).

### 3.10. Protein-Protein Interaction Network

**Figure 5:**
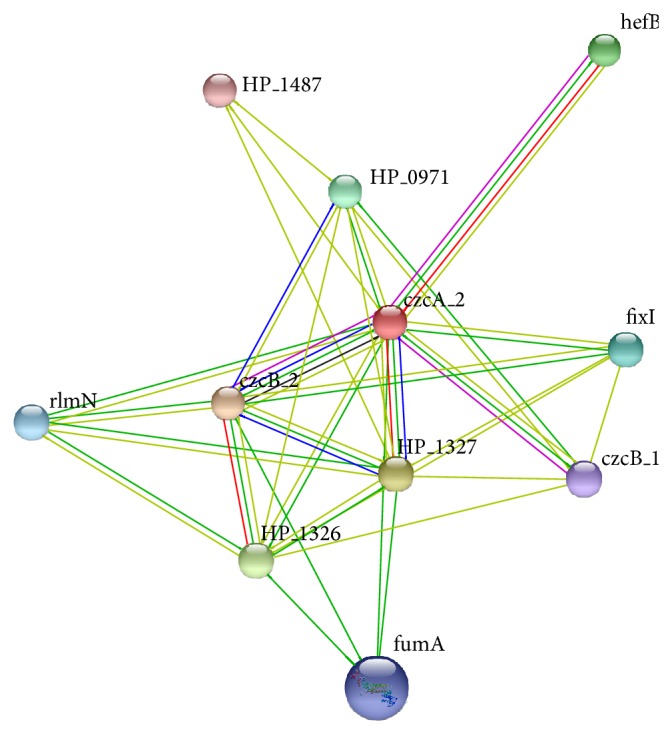
Protein-protein interaction (PPI) map of targeted core proteins. An interactome established between prioritized core protein targets. Eleven proteins showed interactions among each other revealing their collaborations in different pathways. Number and color of edges between nodes represent the type of evidence for the associations.

The 28 predicted potential surface core exoproteins were analyzed to generate a PPI network of* H. pylori*. The interaction map generated by STRING revealed many new insights of protein interactions among target proteins. In principle, the interaction network is constructed by integrating many types of experimentally proved statistics like cooccurrence, neighborhood, gene fusion, homology, text mining, and coexpression [30]. PPI map shown in Figure 5 exhibits direct or indirect relation between targeted proteins; 11 proteins appear to have direct relations/interactions among each other. Further enrichment analyses of proteins appearing in interactome have been carried out to explore potential role of these interactions; KEGG pathway analysis showed involvement of interacting proteins in citrate cycle, metabolic pathways, microbial metabolism in diverse environment, ABC transporters, and various other metabolic pathways. Domain enrichment analysis revealed only 2 proteins having outer membrane efflux protein domain.

### 3.11. Epitope Mapping

The 28 potential proteins were then analyzed for epitopes which can bind to B-cells and generate immune response efficiently. BCPred was used to predict 75% specific 20-mer epitopes of each protein. Each protein has 1 or more sites that have ability to bind B-cells epitopes except a core protein 1028, a glycerol 3-phosphate dehydrogenase. Though it is an enzyme, no epitope was predicted in its sequence [31]. Three core proteins have a single epitope binding site, BCPred suggested 3 proteins with 2 sites for protective antigens, and all other core proteins have >2 epitope binding sites. All predicted epitopes were then subjected to VaxiJen to check their antigenic property (threshold = 0.4, ACC output). Being based on alignment independent method VaxiJen follows autocross covariance (ACC) algorithm that mines protein sequences for antigenicity based on chemical properties [32]. We used mode for bacterial protein prediction for protective antigens. Some proteins' epitopes do not show antigenic behavior whereas as some proteins show highly antigenic epitopes. Complete list of predicted epitopes and their VaxiJen score to be specified as antigenic or not are given in Additional File 5. Epitopes with highest scores for antigenicity can be further checked for MHC class binding and then can be approved as the best targets for vaccine development against* H. pylori*. Some epitopes from core proteins like MEHQNTTQKQGELKRDMKMR residing at position 1 of gene_family = “948” (Additional File 5) have a VaxiJen score of 1.5091, which is much higher than the cutoff value 0.4, whereas some epitopes, for example, MMQLYKKHGANPLGGCLPLI initiating from 407 bp of gene_family = “1486,” has 041 score near to cutoff value. Epitopes with the best value can be subjected to vaccine development. Although there are many loop holes in the defined experimental process of developing therapeutic targets, these* in silico *predicted epitopes can provide a good platform for vaccinology to begin a new era.

### 3.12. Pathogenicity Islands Prediction in* H. pylori*

We have identified 22 nonredundant putative PAIs across the genomes of* H. pylori *strains: 26695, Cuz-20, J99, PeCan4, and SouthAfrica7, which are named as putative pathogenicity islands of* H. pylori* 1–22 (PiHp1–22). To create a heatmap with the percentage of similarity of each PAI in all genomes, the content of PAIs identified as PiHp1–18, PiHp19, PiHp20-21, and PiHp22 were acquired from the genome sequences of* H. pylori *strains 26695, Cuz-20, SouthAfrica7, and PeCan4, respectively. Although some of the reference PAIs may be partially or totally present in the other reference genomes, these reference PAIs were chosen due to the higher representability (according to their size and gene content); for example, PiHp19 in* H. pylori *Cuz20 is also present in* H. pylori *26695; however, it has only 40% of the genes of PiHp19 of* H. pylori *Cuz20 and, thus, the latter one has been chosen for comparative analysis.

**Figure 6:**
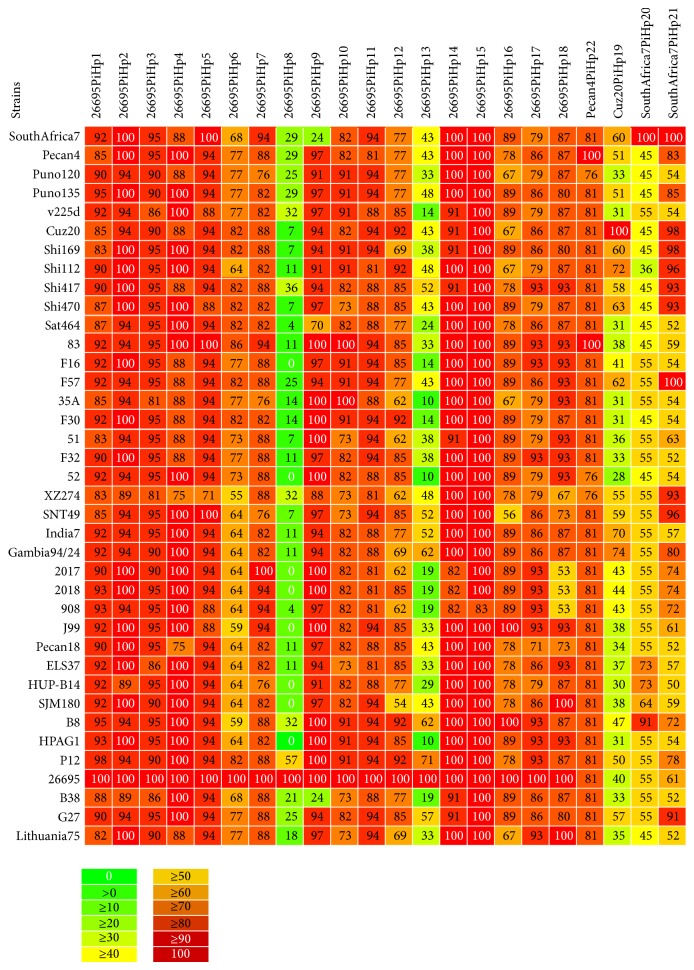
Pathogenicity islands in* H. pylori* genomes (Pan-Heatmap). Heatmap analysis demonstrates high degree of variability on most of the PAIs across all genomes. Among the 22 predicted PAIs, only PiHps 2, 4, 14, and 15 are present in at least 50% of the strains.

**Figure 7:**
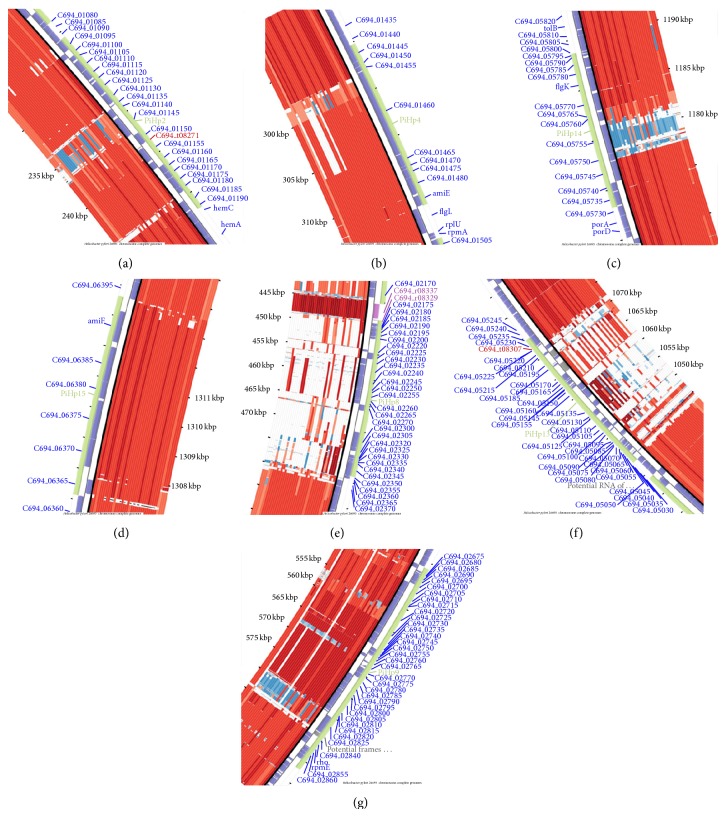
Conserved and variable pathogenicity islands in* H. pylori*. Putative pathogenicity islands predicted in* H. pylori*. The* H. pylori *26695 a reference genome is selected for analysis (scaffold). All the genomes are aligned and a phylogenetically related nonpathogenic organism* Wolinella succinogenes *DSM 1740 is also included for comparison. PAIs 2, 4, 14, and 15 are found conserved ((a), (b), (c), and (d)). On the other side, PAIs PiHps 8, 13, and 9 ((e), (f), and (g)) were found variable among* H. pylori *genomes.

In the heatmap, we observed a high degree of variability in most of the PAIs across all 39 genomes, where only PiHps 2 (C694_01095-C694_01190), PiHps 4 (C694_01445- C694_01490), PiHps 14 (C694_05735-C694_05795), and PiHps 15 (C694_06365-C694_06390) are totally present in at least 50% of the strains (Figure 6). On the other hand, PiHps 8 (C694_02175-C694_02345) and PiHps 13 (C694_05045-C694_05230) are the most variable regions, presenting percentages of similarity ranging from 0–57% to 10–71%, respectively.  Figure 7 demonstrates the conserved PiHps 2, 4, 14, and 15 (Figures 7(a), 7(b), 7(c), and 7(d)) and variable PAIs PiHps 8, 13, and 9 (Figures 7(e), 7(f), and 7(g)).

The majority of the genes harbored by PiHps 2, 4, 8, 13, 14, and 15 have been assigned the term “hypothetical protein” on their product tag, meaning that most of the gene products are not yet identified. However, some genes on these PAIs deserve attention; for example, PiHp2 harbors a gene coding for a DNA repair protein, RadA (C694_01125), which is part of a superfamily of recombinases or DNA strand-exchange proteins, composed of archaeal RadA, bacterial RecA, and eukaryal Rad51 and DMC1 proteins. RadA has a pivotal role in DNA strand-exchange process between single stranded DNA (ssDNA) and a homologous double-stranded DNA (ds-DNA) in homologous recombination [47]. Homologous recombination is one of several DNA repair pathways (direct reversal, base excision repair, nucleotide excision repair, mismatch repair, and recombination repair pathways) and functions in the repair of double-stranded DNA breaks and the restarting of stalled replication forks, therefore, guaranteeing accurate functioning and propagation of genetic information [47, 48]. However, as RadA is mainly found in archaea, in vitro experiments are required to elucidate the putative function of RadA in bacteria, specially* H. pylori* and to clarify its putative acquirement through horizontal gene transfer from archaea.

In PiHp4, there are genes coding for a cell division protein FtsH (C694_01445) and a toxin-like outer membrane protein (C694_01460), also termed putative vacuolating cytotoxin- (VacA) like protein. The cell division protein FtsH is a member of the AAA+ super-family (ATPases associated with diverse cellular activities), which consists of highly conserved molecular machines responsible for a number of cellular processes like cell division, cell differentiation, signal transduction, stress response, and others [49]. FtsH is required for the proper functioning of sigma 54 under nitrogen limitation conditions and FtsH mediated degradation of misassembled membrane and cytoplasmic proteins is thought to be responsible for its role in the heat shock response, therefore explaining its upregulation in response to heat shock in several bacteria, including* H. pylori *[50, 51]. VacA, or vacuolating cytotoxin A, is one of the most studied toxins produced by* H. pylori*, whereas the CagA (cytotoxin-associated gene A) occupies the first place. VacA has the ability to cause vacuole-like membrane vesicles in the cytoplasm of gastric cells and is also associated with disruption of mitochondrial functions, stimulation of apoptosis, and blockade of T-cell proliferation. The presence of the toxigenic allelic s1 form of VacA in strains of* H. pylori *is commonly associated with an increased risk of peptic ulceration and gastric cancer [7, 52–54]. CagA, on the other hand, after injected in host cells, can influence cellular tight junction, cellular polarity, cell proliferation and differentiation, cell scattering, and induction of the inflammatory response. CagA was already identified to be located in an extensively studied pathogenicity island (cag-PAI), which is ~40 kb in size, encodes for a type IV secretion system (T4SS), and was identified by PIPS, in this work, as PiHp9 (C694_02670-C694_02870) (for detailed reviews of VacA and CagA, please refer to [52, 54]).

Inside the most variable PAIs, PiHps 8 and 13, IS605 transposases tnpA (C694_02220, C694_05100 and C694_05150) and tnpB (C694_02225, C694_05105 and C694_05145) can be observed, which are probably responsible for the incorporation of the PAIs and could also account for their high instability. Additionally, PiHp8 also harbors genes coding for 2 VirB4-like proteins (C694_02240 and C694_02345). VirB4 is part of T4SS in bacteria, which is necessary for pilus biogenesis, substrate transfer, and virulence as it is responsible for horizontal transfer of plasmid DNA between bacteria during conjugation and for the delivering of macromolecules to prokaryotes and eukaryotes [55, 56]. In* H. pylori*, the T4SS formed by the VirD4/VirB genes are known to have a pivotal role in the delivery of CagA protein into the cytosol of gastric epithelial cells and the entire T4SS is coded by VirD4/VirB genes harbored by the cag-PAI (PiHp9). The putative backup function of the 2 copies of VirB4-like proteins in PiHp8 for the VirB4 harbored by cag-PAI is yet to be elucidated.

## 4. Conclusion 

The study illustrates the comparative genomic and pan-genomic analysis of important human pathogen* H. pylori. *Multiple genomes from the same species have been analyzed, showing genomic similarities to a large extent, and thus constitute relatively higher conserved core genome. The number of strain specific genes was observed to be significantly low, indicative of the close relationships among these strains. A number of potential novel pathogenicity islands were predicted and vaccine candidates and drug targets have been characterized providing detailed insights into the pathogenesis of* H. pylori*. Therapeutic targets' prioritization includes molecular weight analysis, nonhomology with host, and epitope mapping. Proteins with weight <110 kDa are supposed to be good targets for vaccines as they can easily be extracted and purified. Antigenic epitopes of selected core proteins can provide good insight into development of targeted peptide vaccines. The candidate genes and proteins identified could be further analyzed for the development of therapeutics against this neglected pathogen. Furthermore, conserved pathogenic regions in multiple genomes which have been identified will help in understanding the common pathogenic behavior of this pathogen. Finally, the comparative genomic tools and technique applied in the study can be extended to other pathogens even on a larger scale. We believe that the data generated during this study can assist further research in comparative genome analysis in order to expose the genetic markers of virulence, organism adaptability to host tissues, antibiotic resistance, and effective therapeutic strategies in the same or other similar species.

## Supplementary Material

The supplementary data provides insight view to results originally extracted from different tools and databases. Because of availability of large dataset it was not possible to incorporate all of it the article. You can access the additional files having the composition of core genome families of all 1193 genomes (Additional file 1). Additional file 2 contains BLAST data extracted from DEG, number of homologs along with their AC number against all query proteins. Additional file 3 contains BLAST result of gene families against Human genome to find non-human homologs.
Protein sequence of all gene families has been provided in Additional file 4.
Additional file 5 gives a comprehensive view of all predicted epitopes along with their location and their predictions as antigenic or not.

## Figures and Tables

**Table 1:** List of genomes analyzed in this study. Genomic feature, statistics, accession numbers (chromosome + plasmid) and pangenomic data generated by analyzing 39 *Helicobacter pylori *strains. The number of predicted proteins, clusters, new genes (unique), and core and pan genome is estimated.

Organisms	Genomic features and statistics				Pan-genomics and comparative genomics				
	Size (bp)	Proteins	Chromosome	Plasmid	%AT	New genes	New families	Pan-genome	Core genome
*Helicobacter pylori* 2017	1548238	1503	NC_017374.1		60.698	1503	1491	1491	1491
*Helicobacter pylori* 2018	1562832	1508	NC_017381.1		60.706	29	29	1520	1464
*Helicobacter pylori* 26 695	1667867	1579	NC_000915.1		61.124	207	189	1702	1323
*Helicobacter pylori* 35A	1566655	1500	NC_017360.1		61.132	106	103	1800	1291
*Helicobacter pylori* 51	1589954	1515	NC_017382.1		61.230	80	79	1871	1278
*Helicobacter pylori* 52	1568826	1509	NC_017354.1		61.059	63	62	1926	1273
*Helicobacter pylori* 83	1617426	1559	NC_017375.1		61.277	87	84	2004	1268
*Helicobacter pylori* 908	1549666	1511	NC_017357.1		60.701	24	24	2023	1264
*Helicobacter pylori* B38	1576758	1502	NC_012973.1		60.840	61	52	2065	1237
*Helicobacter pylori* B8 6296	1680029	1579	NC_014256.1	NC_014257.1	61.215	51	50	2097	1235
*Helicobacter pylori* Cuz20	1635449	1536	NC_017358.1		61.136	73	72	2153	1227
*Helicobacter pylori* ELS37	1669876	1564	NC_017063.1	NC_017064.1	61.123	57	55	2194	1224
*Helicobacter pylori* F16	1575399	1508	NC_017368.1		61.115	28	26	2205	1225
*Helicobacter pylori* F30	1579693	1498	NC_017365.1	NC_017369.1	61.197	19	19	2210	1225
*Helicobacter pylori* F32	1581461	1506	NC_017366.1	NC_017370.1	61.143	21	21	2218	1223
*Helicobacter pylori* F57	1609006	1530	NC_017367.1		61.272	16	16	2226	1221
*Helicobacter pylori* G27	1663013	1577	NC_011333.1	NC_011334.1	61.130	51	51	2266	1221
*Helicobacter pylori* Gambia 9424	1712468	1589	NC_017371.1	NC_017364.1	60.876	28	28	2286	1220
*Helicobacter pylori* HPAG1	1605736	1508	NC_008086.1	NC_008087.1	60.933	32	32	2310	1220
*Helicobacter pylori* HUP-B14	1607584	1507	NC_017733.1	NC_017734.1	60.957	37	35	2339	1219
*Helicobacter pylori* India7	1675918	1572	NC_017372.1		61.102	33	32	2362	1217
*Helicobacter pylori *J99	1643831	1505	NC_000921.1		60.811	18	18	2373	1216
*Helicobacter pylori *Lithuania75	1640673	1555	NC_017362.1	NC_017363.1	61.134	42	42	2401	1212
*Helicobacter pylori* P12	1684038	1587	NC_011498.1	NC_011499.1	61.213	22	22	2411	1206
*Helicobacter pylori* PeCan18	1660685	1543	NC_017742.1		60.981	17	17	2415	1208
*Helicobacter pylori* PeCan4	1638269	1532	NC_014555.1	NC_014556.1	61.089	27	27	2435	1208
*Helicobacter pylori* Puno120	1637762	1532	NC_017378.1	NC_017377.1	61.095	26	26	2452	1206
*Helicobacter pylori* Puno135	1646139	1539	NC_017379.1		61.176	14	14	2463	1205
*Helicobacter pylori* SAfrica7	1679829	1573	NC_017361.1	NC_017373.1	61.575	48	47	2498	1206
*Helicobacter pylori *Sahul64	1644275	1579	ALWV01		61.240	32	30	2513	1205
*Helicobacter pylori* Sat464	1567570	1464	NC_017359.1	NC_017356.1	60.908	15	15	2521	1205
*Helicobacter pylori* Shi112	1663456	1561	NC_017741.1		61.227	10	10	2519	1205
*Helicobacter pylori* Shi169	1616909	1516	NC_017740.1		61.136	6	6	2521	1205
*Helicobacter pylori* Shi417	1665719	1545	NC_017739.1		61.229	14	14	2530	1204
*Helicobacter pylori* Shi470	1608548	1518	NC_010698.2		61.229	7	7	2530	1203
*Helicobacter pylori* SJM180	1658051	1524	NC_014560.1		61.100	15	15	2540	1203
*Helicobacter pylori* SNT49	1610830	1514	NC_017376.1	NC_017380.1	61.004	12	12	2540	1202
*Helicobacter pylori *v225d	1595604	1510	NC_017355.1	NC_017383.1	61.055	15	15	2549	1199
*Helicobacter pylori* XZ274	1656544	1701	NC_017926.1	NC_017919.1	61.427	89	89	2614	1193

**Table 2:** Biological categorization of candidate essential gene families. The *H. pylori *EGFs analyzed by CELLO and PSORTb for prediction of surface proteins. This is followed by calculation of molecular weight of proteins by Expasy tool and Blast2go is used for functional annotation.

Protein	Localization	Mol. weight (Da)	EC number	Sequence description
SeqID: /gene_family=“1495”	Cytoplasmic Outer membrane	76937.16		

SeqID: /gene_family=“1623”	Outer membrane Inner membrane	81336.76	EC 3.6.3.3, EC 3.6.3.4, EC 3.6.3.5	Copper-exporting ATPase

SeqID: /gene_family=“1487”	Extracellular Outer membrane	51589.98	EC 3.4.21	Serine protease

SeqID: /gene_family=“1447”	Outer membrane Cytoplasmic	24108.16		

SeqID: /gene_family=“821”	Inner membrane Outer membrane	26170.79		

SeqID: /gene_family=“222”	Inner membrane Cytoplasmic	61901.64		ABC transporter permease

SeqID: /gene_family=“197”	Inner membrane	32817.21	EC 2.5.1	Prenyltransferase

SeqID: /gene_family=“577”	Inner membrane	25149.00		Membrane protein

SeqID: /gene_family=“1486”	Inner membrane	62563.95		Insertase

SeqID: /gene_family=“948”	Inner membrane	52460.09		Lysine-specific permease

SeqID: /gene_family=“1028”	Cytoplasmic Inner membrane	33346.74	EC 1.1.1.8	Glycerol-3-phosphate dehydrogenase

SeqID: /gene_family=“1439”	Inner membrane	46137.77		Potassium transporter

SeqID: /gene_family=“1480”	Inner membrane	53997.01		Sodium proline symporter

SeqID: /gene_family=“714”	Inner membrane	49579.70		Sodium: neurotransmitter symporter family protein

SeqID: /gene_family=“1238”	Inner membrane	115481.83		Cation efflux system protein

SeqID: /gene_family=“230”	Outer membrane Extracellular	30259.72	EC 1.3.1.9	Enoyl-acp reductase

SeqID: /gene_family=“940”	Extracellular	33739.71		

SeqID: /gene_family=“792”	Extracellular	39410.96		

SeqID: /gene_family=“747”	Cytoplasmic Outer membrane Extracellular	32881.19		Domain protein

SeqID: /gene_family=“30”	Periplasmic	30271.1		50s ribosomal protein l2

SeqID: /gene_family=“1043”	Periplasmic Cytoplasmic	15669.30		

SeqID: /gene_family=“128”	Periplasmic Cytoplasmic	37289.58	EC 3.5.1.49	Formamidases

SeqID: /gene_family=“428”	Periplasmic	40467.09	EC 1.1.1.1	NADP-dependent alcohol dehydrogenase

SeqID: /gene_family=“664”	Cytoplasmic Periplasmic	26515.58	EC 3.6.1.3	Abc transporter ATP-binding protein

SeqID: /gene_family=“928”	Periplasmic	61103.25	EC 2.3.2.2	Gamma-glutamyltranspeptidase

SeqID: /gene_family=“1097”	Periplasmic	65613.27	EC 3.1.4.16	5-nucleotidase protein

SeqID: /gene_family=“88”	Cytoplasmic membrane	21533.23		Membrane protein

SeqID: /gene_family=“1595”	Cytoplasmic membrane	74214.44	EC 2.41.129	Penicillin-binding protein 1a

SeqID: /gene_family=“387”	Periplasmic	62296.56		Peptide ABC transporter substrate-binding protein
