# Supplementary material for: Pan-Genome Analysis of Human Gastric Pathogen H. pylori: Comparative Genomics and Pathogenomics Approaches to Identify Regions Associated with Pathogenicity and Prediction of Potential Core Therapeutic Targets
Source: Biomed Res Int. 2015 Jan 29;2015:139580. doi: 10.1155/2015/139580 (PMC4325212; doi:10.1155/2015/139580)
Supplement: Supplementary file 1 — The supplementary data provides insight view to results originally extracted from different tools and databases. Because of availability of large dataset it was not possible to incorporate all of it the article. You can access the additional files having the composition of core genome families of all 1193 genomes (Additional file 1). Additional file 2 contains BLAST data extracted from DEG, number of homologs along with their AC number against all query proteins. Additional file 3 contains BLAST result of gene families against Human genome to find non-human homologs. Protein sequence of all gene families has been provided in Additional file 4. Additional file 5 gives a comprehensive view of all predicted epitopes along with their location and their predictions as antigenic or not. [file 139580.f1.zip › supplementary material/Additional figure 1.pdf]

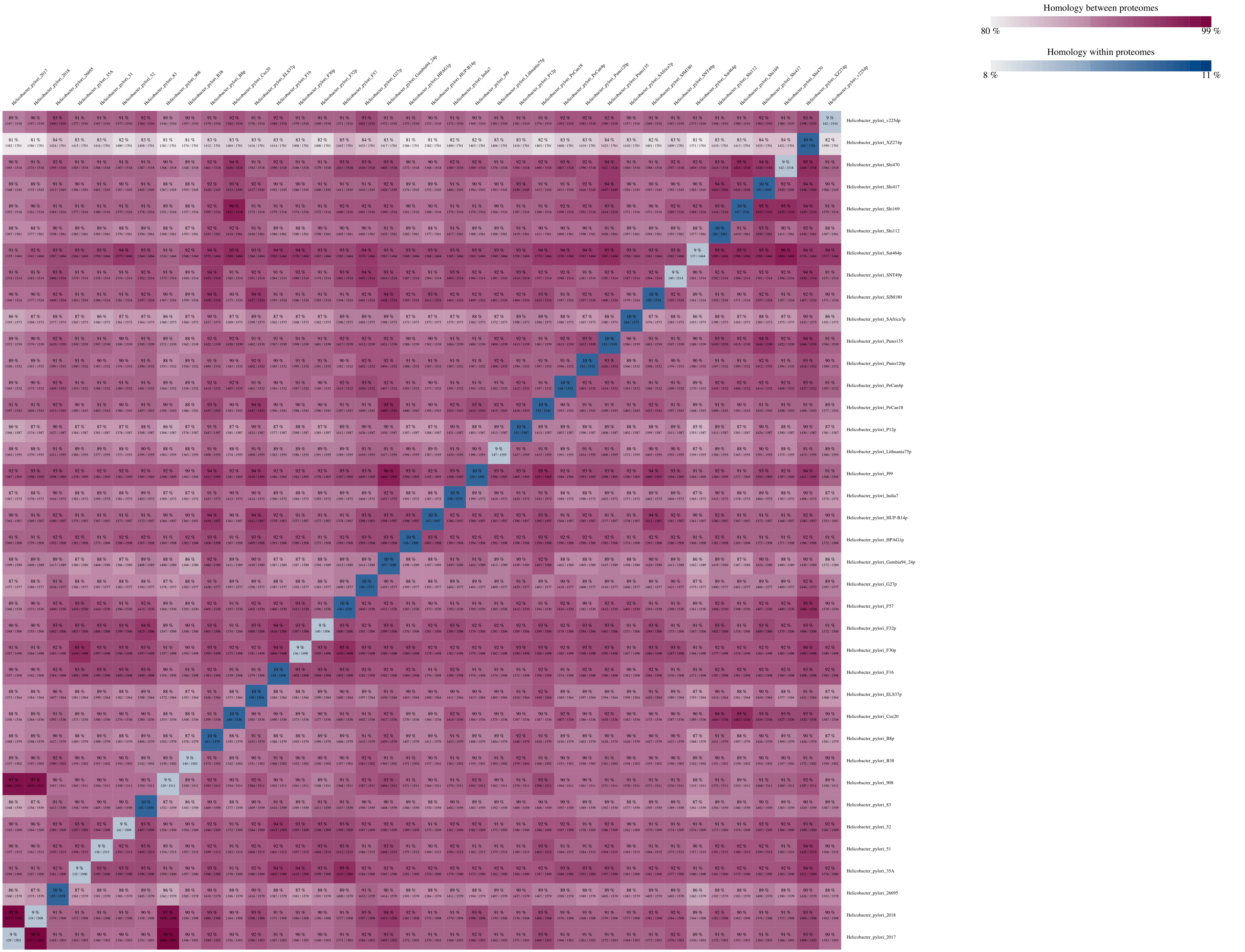
Homology between proteomes
80 %
99 %
Homology within proteomes
8 %
11 %
Helicobacter_pylori_v225dp
Helicobacter_pylori_XZ274p
Helicobacter_pylori_Shi470
Helicobacter_pylori_Shi417
Helicobacter_pylori_Shi169
Helicobacter_pylori_Shi112
Helicobacter_pylori_Sat464p
Helicobacter_pylori_SNT49p
Helicobacter_pylori_SJM180
Helicobacter_pylori_SAfrica7p
Helicobacter_pylori_Puno135
Helicobacter_pylori_Puno120p
Helicobacter_pylori_PeCan4p
Helicobacter_pylori_PeCan18
Helicobacter_pylori_P12p
Helicobacter_pylori_Lithuania75p
Helicobacter_pylori_J99
Helicobacter_pylori_India7
Helicobacter_pylori_HUP-B14p
Helicobacter_pylori_HPAG1p
Helicobacter_pylori_Gambia94_24p
Helicobacter_pylori_G27p
Helicobacter_pylori_F57
Helicobacter_pylori_F32p
Helicobacter_pylori_F30p
Helicobacter_pylori_F16
Helicobacter_pylori_ELS37p
Helicobacter_pylori_Cuz20
Helicobacter_pylori_B8p
Helicobacter_pylori_B38
Helicobacter_pylori_908
Helicobacter_pylori_83
Helicobacter_pylori_52
Helicobacter_pylori_51
Helicobacter_pylori_35A
Helicobacter_pylori_26695
Helicobacter_pylori_2018
Helicobacter_pylori_2017
